# Supplementary material for: gEVAL — a web-based browser for evaluating genome assemblies
Source: Bioinformatics. 2016 Apr 7;32(16):2508–10. doi: 10.1093/bioinformatics/btw159 (PMC4978925; doi:10.1093/bioinformatics/btw159)
Supplement: Supplementary Data [file supp_32_16_2508__index.html]

gEVAL — a web-based browser for evaluating genome assemblies — gEVAL — a web-based browser for evaluating genome assemblies — Supplementary Data 

# gEVAL — a web-based browser for evaluating genome assemblies

## Supplementary Data

files

- Supplementary Data - doc file
