# Supplementary material for: gEVAL — a web-based browser for evaluating genome assemblies
Source: Bioinformatics. 2016 Apr 7;32(16):2508–10. doi: 10.1093/bioinformatics/btw159 (PMC4978925; doi:10.1093/bioinformatics/btw159)

**Supplementary Material – Table 1**

Hosted assemblies in the gEVAL browser as of January 6, 2016.

† - patch releases included

| Species | Assemblies in gEVAL | Description | Source |
| --- | --- | --- | --- |
| Human | GRCh38.p5 † | GRC Reference Assembly | GCA_000001405.20 |
|  | GRCh37.p13 † | GRC Reference Assembly | GCA_000001405.25 |
|  | NCBI36 | IHGSC Reference Assembly | GCF_000001405.12 |
|  | NA12878 | Coriell NA12878 (Broad Institute-ALLPATHS) | AEKP00000000.1 |
|  | CHM1_1.1 | Reference-guided CHM1htert | AMYH00000000.2 |
|  | CHM1htert | CHM1 cell line for PacBio P5C3 sequencing | GCA_000772585.3 |
|  | HuREF | J. Craig Venter assembly | ABBA00000000.1 |
|  | YH2.0 | BGI-YanHuang | GCA_000004845.2 |
|  | Mongolian | BGI-Mongolian Genome | PRJNA258575 |
|  | HS1011v1 | CMT-001 (Charcot-Marie-Tooth Disease) | GCA_001292825.1 |
|  | ASM101398v1 | Coriell NA12878 (Mt. Sinai) | GCA_001013985.1 |
|  | HG003- NA24149 | Ashkenazim Trio – Father (GIAB) | PRJNA200694 |
|  | HG004- NA24143 | Ashkenazim Trio – Mother (GIAB) | PRJNA200694 |
|  | HG002- NA24385 | Ashkenazim Trio – Son (GIAB) | PRJNA200694 |

| Species | Assemblies in gEVAL | Description | Source |
| --- | --- | --- | --- |
| Mouse | GRCm38.p3 † | GRC Reference Assembly | GCA_000001635.5 |
|  | MGSCv37 | GRC Reference Assembly | GCA_000001635.1 |
|  | MmusALLPATHS2 | ALLPATHS C57BL/6J (Broad Institute) | GCA_000185105.2 |
|  | Mm_Celera | Mixed strained WGS assembly | GCA_000002165.1 |
|  | MGSCv3 | House mouse WGS assembly | GCF_000001635.12 |
|  | 129S1/SvImJ | Mouse Genomes Project Strain | ERS076385 |
|  | A/J | Mouse Genomes Project Strain | ERS075416, ERS138733, ERS212195 |
|  | AKR/J | Mouse Genomes Project Strain | ERS075418, ERS212196, ERS154382 |
|  | BALB/cJ | Mouse Genomes Project Strain | ERS076386 |
|  | C3H/HeJ | Mouse Genomes Project Strain | ERS076383 |
|  | C57BL/6NJ | Mouse Genomes Project Strain | ERS076384 |
|  | CAST/EiJ | Mouse Genomes Project Strain | ERS076381 |
|  | CBA/J | Mouse Genomes Project Strain | ERS076379 |
|  | DBA/2J | Mouse Genomes Project Strain | ERS661327 |
|  | FVB/NJ | Mouse Genomes Project Strain | ERP000687 |
|  | LP/J | Mouse Genomes Project Strain | ERS076382 |
|  | NOD/ShiLtJ | Mouse Genomes Project Strain | ERS076389 |
|  | NZO/HlLtJ | Mouse Genomes Project Strain | ERS076387 |
|  | PWK/PhJ | Mouse Genomes Project Strain | ERS076378 |
|  | SPRET/EiJ | Mouse Genomes Project Strain | ERS076388, ERS138732 |
|  | WSB/EiJ | Mouse Genomes Project Strain | ERS076380 |
|  | CAROLI/EiJ | Mouse Genomes Project Strain | Unpublished, D. Thybert |
|  | Pahari/EiJ | Mouse Genomes Project Strain | Unpublished. D. Thybert |

| Species | Assemblies in gEVAL | Description | Source |
| --- | --- | --- | --- |
| Zebrafish | GRCz10 | GRC Reference Assembly | GCA_000002035.3 |
|  | Zv9 | WTSI Reference Assembly | GCA_000002035.2 |
|  | WGS28 | Tuebingen WGS assembly | Unpublished, Z. Ning |
|  | WGS29 | Tuebingen WGS assembly | Unpublished, Z. Ning |
|  | WGS31 | Tuebingen WGS assembly | GCA_000767325.1 |
|  | WGS32 | Tuebingen WGS assembly | PRJEB11588 |

| Species | Assemblies in gEVAL | Description | Source |
| --- | --- | --- | --- |
| Pig | Sscrofa10.2 | SGSC Reference Assembly | GCA_000003025.4 |

| Species | Assemblies in gEVAL | Description | Source |
| --- | --- | --- | --- |
| Chicken | Galgal5.0 | ICGC Reference Assembly | GCA_000002315.3 |
|  | Galgal4.0 | ICGC Reference Assembly | GCA_000002315.2 |
|  | Galgal4.1_pb | ICGC assembly | Unpublished, D. Burt |

| Species | Assemblies in gEVAL | Description | Source |
| --- | --- | --- | --- |
| Rat | Rnor5.0 | RGSC Assembly | GCA_000001895.3 |

| Species | Assemblies in gEVAL | Description | Source |
| --- | --- | --- | --- |
| Helminths | *Echinococcus multilocularis* | WTSI Helminth Project | WTSI Data Download ‡ |
|  | *Schistosoma mansoni* | WTSI Helminth Project | WTSI Data Download ‡ |
|  | *Stronglyoides ratti* | WTSI Helminth Project | WTSI Data Download ‡ |

‡ http://www.sanger.ac.uk/resources/downloads/helminths/

**Supplementary Material –Table 2**

Single molecule optical and genome maps available in gEVAL as of January 6, 2016.

| Platform | Species | Map | Source |
| --- | --- | --- | --- |
| Bionano Genomics | Human | CHM1 | McDonnell Genome Institute |
|  |  | NA12878 | Genome in a Bottle |
|  |  | HG002-NA24385 | Genome in a Bottle |
|  |  | HG003-NA24149 | Genome in a Bottle |
|  |  | HG004-NA24143 | Genome in a Bottle |
|  |  | HG005-NA24631 | Genome in a Bottle |
|  | Zebrafish | SAT (ZDB-GENO-100413-1) | Wellcome Trust Sanger Institute |
|  | Mouse | PWK/PhJ | Wellcome Trust Sanger Institute |
|  |  | BALB/cJ | Wellcome Trust Sanger Institute |
|  |  | AKR/J | Wellcome Trust Sanger Institute |
|  |  | A/J | Wellcome Trust Sanger Institute |
|  |  | FVB/NJ | Wellcome Trust Sanger Institute |
|  |  | C3H/HeJ | Wellcome Trust Sanger Institute |
|  |  | SPRET/EiJ | Wellcome Trust Sanger Institute |
| OpGen | Mouse | CAST/EiJ | Wellcome Trust Sanger Institute |
|  |  | SPRET_EiJ | Wellcome Trust Sanger Institute |
|  | Chicken | Galgal4 | Wellcome Trust Sanger Institute |
| Schwartz Optical Mapping | Human | GM10860 | David Schwartz (University of Wisconsin) |
|  |  | GM15510 | David Schwartz (University of Wisconsin) |
|  |  | GM18994 | David Schwartz (University of Wisconsin) |
|  | Mouse | C57Bl/6J | David Schwartz (University of Wisconsin) |

**Supplementary Material – Figure 1**

An extended comparative view of a region in GRCh38 chromosome 11 (48,826,475-48,995,020) against 4 other human assemblies. HuREF, YH2, Mongolian, HS1011v1, ASM101398v1 (top to bottom). Comparative analysis reveals missing sequence of roughly 7-8kb within clone AC027369 around ~48,918,942bp of chromosome 11, but present in all other assemblies (A). Furthermore, the region between roughly 48,881,657-48,898,010 appears to be a large inverted duplication (B).


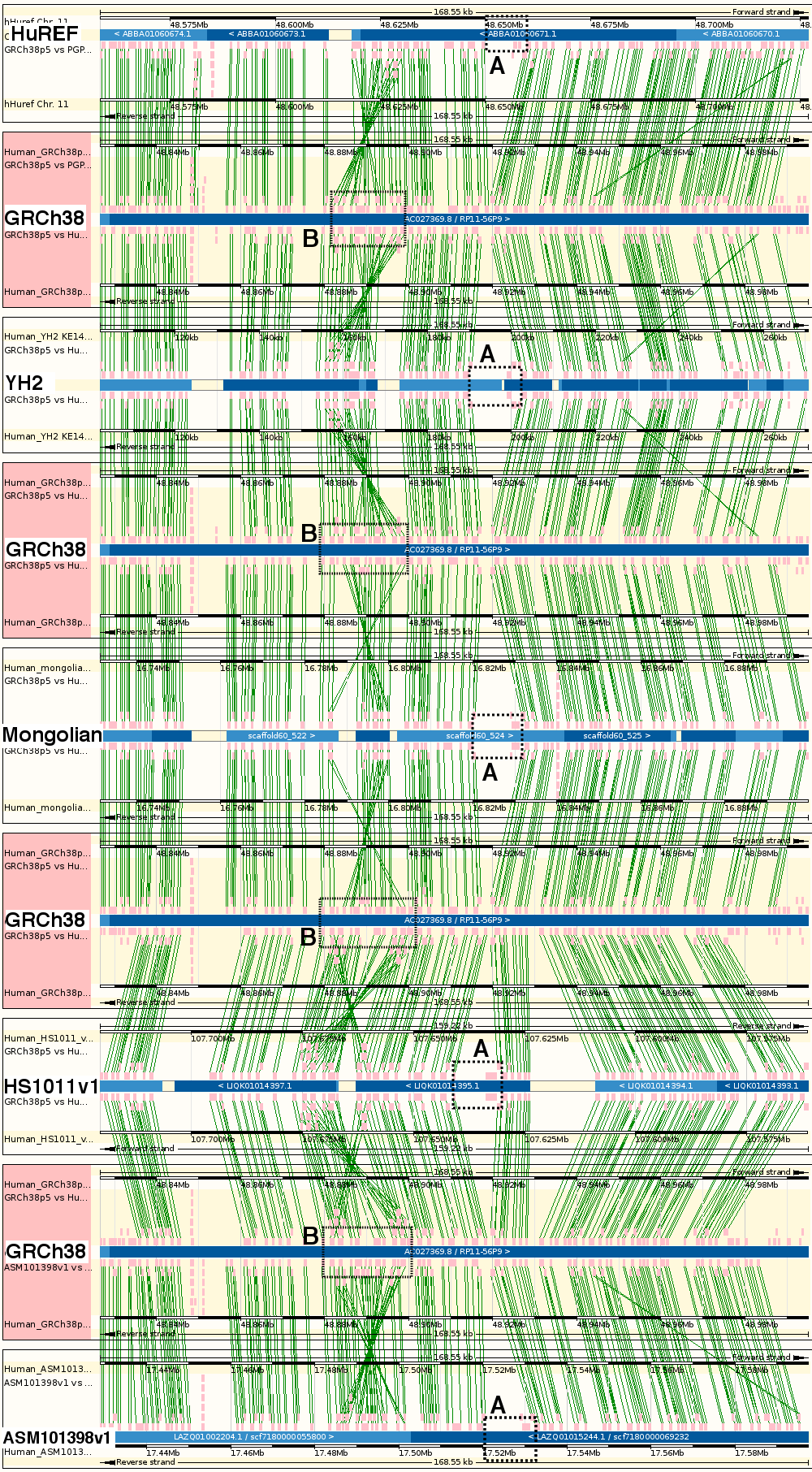

Supplement: Supplementary Data [file supp_btw159_supplementary.doc]
